# Molecular and Clinical Significance of Fibroblast Growth Factor 2 in Development and Regeneration of the Auditory System

**DOI:** 10.3389/fnmol.2021.757441

**Published:** 2021-12-23

**Authors:** Minjin Jeong, Katarina Bojkovic, Varun Sagi, Konstantina M. Stankovic

**Affiliations:** ^1^Department of Otolaryngology—Head and Neck Surgery, Stanford University School of Medicine, Stanford, CA, United States; ^2^Department of Otolaryngology—Head and Neck Surgery, Massachusetts Eye and Ear and Harvard Medical School, Boston, MA, United States; ^3^University of Minnesota Medical School, Minneapolis, MN, United States

**Keywords:** FGF2, hearing loss, tympanic membrane repair, auditory development, vestibular schwannoma

## Abstract

The fibroblast growth factor 2 (FGF2) is a member of the FGF family which is involved in key biological processes including development, cellular proliferation, wound healing, and angiogenesis. Although the utility of the FGF family as therapeutic agents has attracted attention, and FGF2 has been studied in several clinical contexts, there remains an incomplete understanding of the molecular and clinical function of FGF2 in the auditory system. In this review, we highlight the role of FGF2 in inner ear development and hearing protection and present relevant clinical studies for tympanic membrane (TM) repair. We conclude by discussing the future implications of FGF2 as a potential therapeutic agent.

## Introduction

Fibroblast growth factors (FGFs) comprise a large family of proteins which are involved in several biological functions including embryonic development, cell growth and differentiation, angiogenesis, and wound healing (Beenken and Mohammadi, 2009). Various experimental approaches including tissue ablation, transplantation, and development of knockout animals have revealed the important function of FGF signaling in auditory development and function. Several members of the FGF gene family and its receptors have been found to control cell proliferation and specification during the development of sensory progenitors (Huh et al., 2012, 2015; Mansour et al., 2013; Ono et al., 2014) and the inner ear sensory epithelia (Vendrell et al., 2000; Carnicero et al., 2004).

FGF2 binds and activates FGF receptors (FGFRs) primarily through the RAS-mitogen activated protein kinase (MAPK) pathway to regulate cellular function in the skin, blood vessels, tendons, ligaments, bone, and teeth (Beenken and Mohammadi, 2009; Yun et al., 2010; Park et al., 2017). FGF2 is present in human spermatozoa and exposure to recombinant FGF2 has been used to improve recovery of sperm motility (Garbarino Azúa et al., 2017). A role for FGF2 has been described in learning and memory (Graham and Richardson, 2011), neuropsychiatric disorders including anxiety (Perez et al., 2009; Eren-Koçak et al., 2011; Turner et al., 2012; Salmaso et al., 2016), and depression (Mallei et al., 2002; Maragnoli et al., 2004; Riva et al., 2005; Elsayed et al., 2012; Turner et al., 2012; Tang et al., 2017), stress-related disorders (Molteni et al., 2001a, b; Fumagalli et al., 2005; Xia et al., 2013), and schizophrenia (Klejbor et al., 2006; Terwisscha van Scheltinga et al., 2010). Increased levels of FGF2 have been shown to have positive effects in models of neurodegenerative diseases such as Parkinson’s disease (Claus et al., 2004; Timmer et al., 2007), Alzheimer’s disease (Cummings et al., 1993; Kiyota et al., 2011), multiple sclerosis (Ruffini et al., 2001), and traumatic brain injury (Sun et al., 2009; Thau-Zuchman et al., 2012). In contrast, it has been suggested that FGF2 may be a positive regulator for nicotine, amphetamine, cocaine, and alcohol use (Even-Chen and Barak, 2019). These early studies suggest that the role of FGF2 may vary based on the disease context. Nevertheless, there are currently no FDA-approved FGF2 therapies for any pathology.

Despite the discoveries indicating the growing importance of FGF2, there is a lack of complete understanding of FGF2’s role in the auditory system. In this review, we summarize the literature on FGF2 in the auditory system including its function in inner ear development, role in hearing protection, and application in clinical trials of tympanic membrane (TM) reconstruction.

## FGF2 in Auditory Development and Maintenance

**Figure 1:**
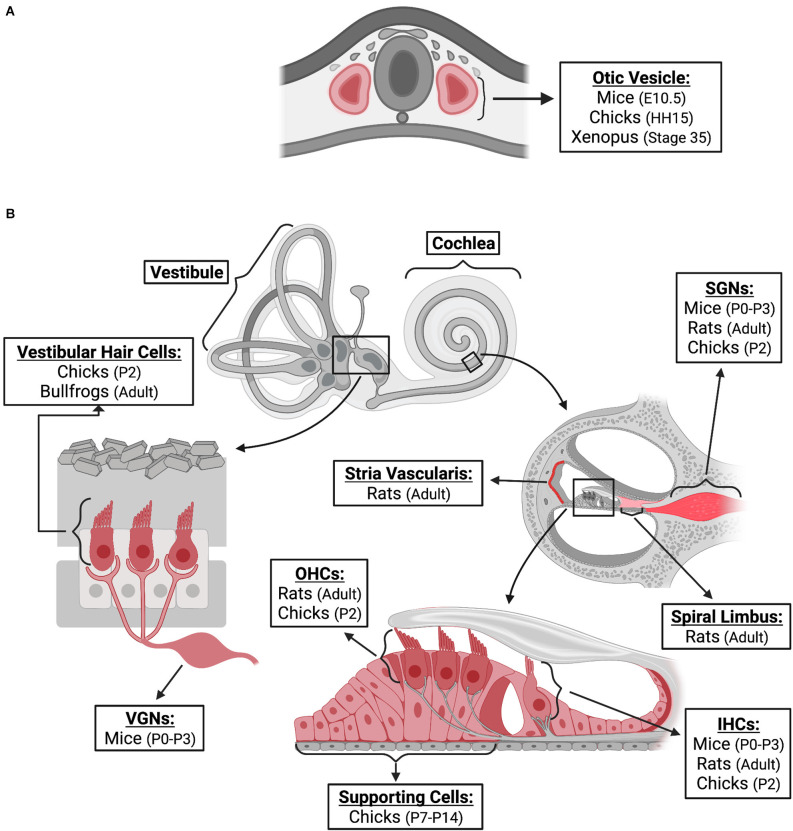
FGF2 expression in inner ear. FGF2 expression locations are indicated in red. The species, and age at which expression was confirmed, are provided for different locations of the inner ear during embryonic development **(A)** and post-natal **(B)** stages. Locations of expression are also provided for non-mammalian models; however, the depiction is based on similar structures of the mammalian inner ear. FGF2, fibroblast growth factor 2; E, embryonic; HH, hamburger-hamilton; P, post-natal; VGNs, vestibular ganglion neurons; SGNs, spiral ganglion neurons; OHCs, outer hair cells; IHCs, inner hair cells. Created with BioRender.com.

FGF2 has been proposed to fulfill many functions during vertebrate auditory development. The expression of FGF2 has been detected through messenger RNA (mRNA) and protein levels in the cochlea and vestibule throughout the life span of animal models (Figure 1). The precise locations of expression are described in the following paragraphs. Furthermore, several studies have examined the impact of exogenously added FGF2 on various developmental stage models (Table 1).

**Table 1:** Studies of exogenous FGF2 in developmental stage models.

Study	Species	FGF2 Dosage (ng/ml)	Outcome
**Mammalian models**			
Hossain and Morest (2000)	Cochlear ganglion from E11 mice	25	FGF2 enhanced migration and early neurite outgrowth
Bruno et al. (2017)	Murine auditory neuroblasts derived from ventral otocyst	25	Induce proliferation and survival depending on Sphingosine 1-phosphate metabolism
Mueller et al. (2002)	Cochelar explant from E13 mice	300	Increase in the number of pillar cells and inner hair cells.
**Non-mammalian models**			
Hossain et al. (1996)	Otocysts from White Leghorn chicken embryos at HH stages 14–16	0.25–10	Increase explant growth, neuroblast migration, and neurite outgrowth 2–10-fold
Zhou et al. (1996)	Rhombic lip from white leghorn chicken embryos at E5.5	10	FGF2 stimulates neurite outgrowth in the cochlear and vestibular nuclei
Lombardo and Slack (1998)	Neural plate stage of Xenopus embryo	10,000	Induce the formation of ectopic otic vesicles
Adamska et al. (2001)	Developing chicken inner ear at HH stages 10–11	FGF2 soaked beads	FGF2 induces ectopic structures that express ear marker genes such as *SOHo1, cNkx5–1*
Carnicero et al. (2001)	Cochlear and vestibular neurons from chicken embryos	0–10	FGF2 stimulated survival of isolated cochlear and vestibular neurons. Overexpression of FGF2 in cochlear neurons resulted in neuronal differentiation
Carnicero et al. (2004)	Sensory epithelial cells from chicken embryos at stage 34	Overexpression of FGF2 by viral vectors	Increases the number of cells expressing early hair cell markers during embryonic development, but did not promote cell proliferation

*E, embryonic day; HH, Hamburger-Hamilton*.

### Mammalian Models

#### Endogenous FGF2 Expression

In mice, FGF2 protein was expressed as early as the embryonic day (E)-10.5 stage (when the otocyst is closed as a sac) of inner ear development in the otocyst epithelium and neuroepithelium (Frenz et al., 1994). Over the period E10.5–E15.5, *FGF2* mRNA expression showed large increases of ~10^3^ fold, and this expression was relatively evenly distributed throughout the mouse cochlea (lateral wall, the center of modiolus, sensory/neural area) except in the cartilage where it was low in the postnatal day (P)-0 mouse cochlea (Pickles, 2001). During the neonatal period of mice (P0-P3), FGF2 immunoreactivity was present in inner ear hair cells, spiral ganglion neurons (SGNs), vestibular ganglion neurons (VGNs), and the auditory brainstem (Després et al., 1991). In the adult stage of rats, FGF2 immunoreactivity was observed in the inner (IHCs) and outer hair cells (OHCs) of the organ of Corti, SGNs, spiral limbus, and stria vascularis (Silva et al., 2005). In the central auditory pathways, FGF2 immunoreactivity was found in the cytoplasm of the neurons of the cochlear nuclei, trapezoid body nuclei, medial geniculate nucleus, and inferior colliculus (Silva et al., 2005). These studies demonstrate that FGF2 can play an important role in embryonic and postnatal auditory development and may also influence the maintenance of adult auditory structure and function.

However, these observations conflict with findings that *FGF2* mRNA was undetectable in the rat cochlea at any age (E 16—*P* > 60; Luo et al., 1993), and that FGF2-like protein was widely distributed in the auditory brainstem but not found in the adult rat cochlea (Després et al., 1991). It is possible that immunostaining of FGF2 observed in adult rat cochlea represented FGF1 immunoreactivity as FGF1 shares 55% homology with FGF2 and they have similar physiological actions and interact with the same receptors. However, this is less likely as the absorption of the FGF2 antibody by FGF1 did not modify the pattern of FGF2 immunoreactivity (Silva et al., 2005). Also, the specificity of the immunoreaction was demonstrated by the complete disappearance of immunoreactivity in the central and peripheral auditory pathways after absorption of FGF2 antibody by FGF2 protein (Silva et al., 2005). These contradicting results may reflect a loss of antigenicity due to decalcification which may have impaired proper localization of endogenous FGF2 mRNA and protein in the cochlea. Additionally, these discrepancies may be related to species or strain differences. For example, Silva et al. (2005) utilized Wistar rats whereas both Després et al. (1991) and Luo et al. (1993) utilized Sprague-Dawley rats. Furthermore, Silva et al. (2005) used male rats whereas gender was not specified in the other two studies. Only one study (Luo et al., 1993) explored the level of FGF2 expression in embryonic stage rats. Comparing these findings with embryonic studies done in mice likely introduces confounding factors related to species differences.

#### Impact of Exogenous FGF2

When FGF2 was added to the cochlear ganglion of E11 stage mice, it enhanced the migration and initial differentiation of cochlear ganglion neurons (Hossain and Morest, 2000). In addition, FGF2 was found to stimulate the proliferation of mouse auditory neuroblasts and protected these cells from apoptosis (Bruno et al., 2017). Interestingly treatment with FGF2 also led to a greater than two-fold increase in the number of pillar cells (located in the region between the single row of IHCs and the first row of OHCs) and to a small increase in the number of IHCs (Mueller et al., 2002).

### Non-mammalian Models

#### Endogenous FGF2 Expression

During inner ear development in chicken, weak expression of FGF2 was observed in the otic placode, neural tube, and notochord at stage Hamburger-Hamilton (HH)-11 (40–45 h). Expression was increased during the formation of the otic vesicle at HH15 (50–55 h; Vendrell et al., 2000). *FGF2* mRNA expression was detected in the otocyst at E5 and in the cochlea, cochlear nerve ganglion, cochlear hair cells, and vestibular hair cells at P2 (Pickles and van Heumen, 1997). FGF2 expression begins early, and thus, it is thought to regulate cell proliferation. At the young chick stage, FGF2 was localized in the nuclei of supporting cells throughout the entire length of the basilar papilla, but not hair cells (Lee and Cotanche, 1996). This suggests that supporting cells have the potential to proliferate *via* a signaling pathway involving FGF2 (Lee and Cotanche, 1996). In the pre-larval Xenopus embryo (stage 35), FGF2 protein was expressed in the otic vesicle (Song and Slack, 1994). In the adult stage of bullfrogs, vestibular hair cells expressed FGF2 and the receptors FGFR1 and FGFR2 while supporting cells did not express either molecule (Cristobal et al., 2002).

#### Impact of Exogenous FGF2

The addition of FGF2 has been found to promote explanted cell culture and neurite growth as well as increase neuroblast migration in otocysts extracted from chick embryos (Hossain et al., 1996). It has also been shown to stimulate neurite outgrowth in the cochlear and vestibular nuclei of chick embryos (Zhou et al., 1996). In addition, when applied *via* heparin beads at the neural plate stage in the Xenopus embryo, FGF2 was found to induce the formation of ectopic otic vesicles (Lombardo and Slack, 1998). Furthermore, when introduced in the developing chick inner ear, it induced ectopic structures that expressed ear marker genes and increased the size of the vestibulo-cochlear ganglion (Adamska et al., 2001). It was also found to stimulate survival of isolated chicken embryonic cochlear and vestibular neurons and to promote neuronal differentiation (Carnicero et al., 2001). In sensory epithelial cells from chicken embryos, the addition of FGF2 increased the number of cells expressing early hair cell markers but did not promote cell proliferation (Carnicero et al., 2004).

The current studies from vertebrate models lend support to the important role of FGF2 in auditory development. However, further investigation is required to better understand the precise spatial distribution and chronological sequence of FGF2 expression in ear development. Future studies must carefully consider the role of animal species, age, and environmental conditions in the experimental design.

## FGF2 in Inner Ear Cell Survival, Proliferation, and Differentiation

The effect of FGF2 on post-development stage sensory epithelial and neural cells *in vitro* has been well characterized (Table 2). FGF2 was found to promote survival and neuritogenesis of adult rat auditory neurons (Lefebvre et al., 1991) and to significantly enhance the proliferation of rat utricular supporting cells (Zheng et al., 1997). Furthermore, FGF2 stimulated immortalized rat utricular epithelial cell proliferation and differentiation into cells expressing early hair cell markers (Zheng et al., 1998). FGF2 also increased P5 rat Schwann cell proliferation (Hansen et al., 2001). Following mechanical changes induced during isolation and dissociation procedures, FGF2 strongly promoted regeneration of murine SGNs *via* FGFR-3-IIIc receptor (Wei et al., 2007). In acoustic ganglia extracted from fully developed chicks, FGF2 was shown to enhance the survival of statoacoustic neurons (Warchol and Kaplan, 1999).

**Table 2:** *In vitro* studies of FGF2 in sensory and neural cell survival, proliferation, and differentiation.

Study	Cell types and Species	FGF2 Dosage (ng/ml)	Outcome	Effect
**Mammalian models**				
Lefebvre et al. (1991)	Acoustic ganglia from adult Wistar rats	125–4,000	FGF2 promotes the survival and neuritogenesis of the adult afferent auditory neurons	Stimulate
Zheng et al. (1997)	Utricular epithelial cells from P4–P5, Wistar rats	0.1–100	FGF2 significantly enhanced the proliferation of the utricular supporting cells	Stimulate
Zheng et al. (1998)	Utricular epithelial cells from P3–4 Wistar rats	100	FGF2 stimulated proliferation and induced cells to undergo morphological differentiation and express early hair cell markers	Stimulate
Hansen et al. (2001)	Schwann cells from P5 rat	10	FGF2 increased Schwann cell proliferation *via* MAPK signaling	Stimulate
Malgrange et al. (2002)	Organ of Corti from adult Dukin-Hartley guinea pigs	1,000–1,000,000	No detectable protective effect on OHC survival	None
Wei et al. (2007)	SGNs from adult mice (2–3 months of age)	10	FGF2 strongly promoted neurite regeneration	Stimulate
Rak et al. (2014)	Cochlear nucleus neurons from P6 Bl6 mice	10	No effect on overall cell growth, the number of neurons, and the ratio of neurons per cell	None
**Non-mammalian models**				
Warchol and Kaplan (1999)	Acoustic ganglia from chicks	0.1, 1, 10, 100	Enhanced the survival of stato-acoustic neurons	Stimulate

*P, postnatal day; MAPK, mitogen-activated protein kinase; OHC, outer hair cell; SGNs, spiral ganglion neurons*.

Not all studies have shown the stimulatory effect of FGF2 in cell cultures. FGF2 had no detectable influence on the survival of guinea pig OHCs (Malgrange et al., 2002) or dissociated mouse cochlear nucleus neurons (Rak et al., 2014).

## FGF2 as A Protective Or Restorative Agent in Auditory Trauma

The therapeutic potential of FGF2 for induced auditory trauma has been explored in several pre-clinical models in both *in vitro* and *in vivo* experiments (Table 3). Evidence is mixed regarding the degree of effect conferred by FGF2 treatment; however, several studies have shown treatment efficacy. FGF2 has been shown to have both protective and rescue effects. Protective effects signify prevention of auditory damage when given before induced auditory or ototoxic trauma. Rescue effects, conversely, refer to a reversal of auditory damage following an auditory insult.

**Table 3:** FGF2 in studies of induced auditory trauma.

Study	Species	Indication	FGF2 Dosage	FGF2 treatment approach	Outcome	Effect
*In vitro* **Mammalian Models**					
Low et al. (1996)	Organ of Corti of Sprague-Dawley rats	Neomycin induced ototoxicity for 2 days	500 ng/ml	Pre (2 days before) + co-treatment with Neomycin	A greater extent of outer hair cell survival and a significant decrease in stereociliary damage	Preventative
Zheng and Gao (1996)	Cochleae from P3 Wistar rats	Sodium salicylate, gentamicin, and cisplatin- induced ototoxicity	10–100 ng/ml	Co-treatment with ototoxins for 2 days	No protective effects on either SGNs or hair cells	None
Wang et al. (1998)	Spiral ganglion cell of mouse	Glutamate induced ototoxicity for 2 h	25, 50, 100 ng/ml	Post (immediately after)	Increased survival and longer neurites with dose- dependent effect	Rescue
Zhai et al. (2004)	SGNs from P3 mice	Glutamate induced ototoxicity for 2 h	0, 25, 50, 100 ng/ml	Post (immediately after)	Promotion of neurite outgrowth and an increase in the number of surviving SGNs	Rescue
Lou et al. (2014)	Organ of Corti from P3-P5 Wistar rats	Neomycin induced ototoxicity for 2 days	10 ng/ml	Post (immediately after)	No effect on the survival of auditory hair cells and regeneration	None
*In vitro* **Non-Mammalian Models**					
Oesterle et al. (2000)	Utricles or cochlear ducts from P7–P18 white leghorn chickens	Neomycin induced ototoxicity for 1 day	0.01–100 ng/ml	Post (1 day after)	Inhibited cell proliferation but stimulated precursor cell differentiation in inner ear sensory epithelia	None
*In vivo* **Mammalian Models**					
Zhai et al. (1997)	Guinea pigs, (*n* = 19)	Blast-induced hearing loss	-	Post (immediately after)	Average 31 dB improvement in CAP threshold, and less damage of hair cells	Rescue
Yamasoba et al. (2001)	Guinea pigs, (*n* = 22)	Noise-induced trauma	10,000 ng/ml	Pre (4 days before)	No improvement in ABR thresholds or hair cell damage when compared to control	None
Yin et al. (2002)	Guinea pigs, (*n* = 36)	Gentamicin induced ototoxicity	Liposome mediated FGF2/GFP gene transfer into the cochlea	Pre (1 day before)	Average 33 dB improvement in ABR threshold and less damage of hair cells when compared to control	Protective
				Post (8 days after)	Average 29 dB improvement in ABR threshold and less damage of hair cells when compared to control	Rescue
Zhai et al. (2002)	Guinea pigs, (*n* = 15)	Noise-induced trauma	10 μl FGF2 (4000 IU/ml) *via* intracochlear delivery	Post (immediately after)	Average 19 dB improvement in ABR thresholds, and less damage of hair cells	Rescue
Sekiya et al. (2003)	Sprague-Dawley rats	Compression of cochlear neurons	4 μg FGF2 *via* gelatin sponge	Post (immediately after)	Improvement number of SGCs in the basal turn	Rescue
Shi et al. (2003)	Guinea pigs	Noise-induced trauma	IRES- FGF2-GFP plasmid into the round window	Pre (7 days before)	Average 15 dB improvement in ABR threshold when compared to control	Protective
				Post (immediately after)	Average 6 dB improvement in ABR threshold when compared to control	Rescue
Wimmer et al. (2004)	Guinea pigs, (*n* = 30)	Cisplatin induced ototoxicity	7,500 ng	Pre (just before)	No improvement in otoacoustic emissions or outer hair cell damage when compared to control	None
Zhai et al. (2004)	Guinea pigs, (*n* = 20)	Noise-induced trauma	50 IU/100 g FGF2 (4000 IU/ml) *via* intramuscular injection to buttocks, volume not specified	Post (immediately after)	Average 27 dB improvement in ABR thresholds, and less damage of hair cells	Rescue
*In vivo* **Non-Mammalian Models**					
Umemoto et al. (1995)	White leghorn chicks at 4–7 days of age	Acoustic trauma	-	-	FGF2 protein expression was increased in the supporting cells and glial cells near the habenula perforate following acoustic trauma	-
Lee and Cotanche (1996)	White leghorn chicks at 1–2 weeks of age	Acoustic trauma	-	-	No change in the distribution of FGF2 protein in supporting cells following acoustic trauma, no quantification was performed	-
Pickles and van Heumen (1997)	Chicks aged 2–12 days	Gentamicin induced ototoxicity	-	-	No increase in FGF2 mRNA in supporting cells following oto-toxin induced damage	-

*SGNs, spiral ganglion neurons; TM, tympanic membrane; CAP, compound action potential; ABR, auditory brainstem response; GFP, green fluorescent protein; dB, decibel; IRES, internal ribosome entry site; SGCs, spiral ganglion cells; mRNA, messenger RNA. Protective effects signify prevention of auditory damage when given before induced auditory or ototoxic trauma. Rescue effects, conversely, refer to reversal of auditory damage following an auditory insult*.

### *In vitro* Studies

FGF2 was found to protect rat cochlear hair cells in the explanted organ of Corti from aminoglycoside injury when given as a pre-treatment or as a co-treatment with neomycin (Low et al., 1996). FGF2 also had a rescue effect on murine cochlear neurons from glutamate neurotoxicity by promoting neurite outgrowth and increasing the number of surviving SGNs when provided after glutamate application (Wang et al., 1998; Zhai et al., 2004). However, FGF2 was not always effective in ototoxic trauma. For example, FGF2 treatment didn’t affect either SGNs or hair cells when provided as a co- or post-treatment with ototoxins (Zheng and Gao, 1996; Lou X. et al., 2015). Interestingly, in utricles and cochlear ducts of chickens following neomycin-induced ototoxicity, FGF2 was found to inhibit cell proliferation which was not observed in mammals (Oesterle et al., 2000). Therefore, FGF2 may be involved in stimulating precursor cell differentiation as opposed to proliferation in the non-mammal inner ear epithelia.

### *In vivo* Studies

*In vivo* pre-clinical studies of FGF2 have been performed with various delivery methods and animal models and have explored applications in auditory damage and hearing loss. Intramuscular or intracochlear delivery of FGF2 protein significantly improved the hearing threshold and reduced the loss of IHCs after noise exposure in guinea pigs (31, 27, and 19 mean dB improvement respectively when compared to control; Zhai et al., 1997, 2002, 2004). Application of FGF2 also ameliorated degeneration of cochlear nerve in rats after compression (Sekiya et al., 2003). Conversely, delivery of FGF2 prior to noise exposure or cisplatin-induced ototoxicity had no protective effect in guinea pigs (Yamasoba et al., 2001; Wimmer et al., 2004).

In addition, FGF2 gene therapy has also been investigated as a potential treatment modality. Administration of an FGF2 genetic construct *via* the internal ribosome entry site-FGF2-green fluorescent protein (IRES-FGF2-GFP) plasmid had a protective and rescue effect (15 and 6 mean dB improvement respectively when compared to control) following noise-induced trauma in guinea pigs (Shi et al., 2003). Additionally, liposome-mediated FGF2 gene transfer into the cochlea of guinea pigs had a similar protective and rescue (33 and 29 mean dB improvement respectively when compared to control) effect on hearing from gentamicin-induced ototoxicity (Yin et al., 2002).

Furthermore, endogenous FGF2 expression following acoustic trauma was studied in non-mammalian models. In contrast to mammalian models, non-mammalian vertebrates are capable of hair cell regeneration. Lee and Cotanche (1996) described an identical distribution pattern of FGF2 in supporting cells with exclusion from hair cells in both noise-exposed and control chicks, but they did not quantitatively evaluate expression levels. A separate study showed that FGF2 protein expression levels were increased in the supporting cell layer following noise-induced damage of the chick inner ear (Umemoto et al., 1995). Taken together, these results suggest that FGF2 may play a role in hair cell regeneration from supporting cells. Since regenerative processes are likely to recapitulate developmental processes, it is not surprising that regeneration appears to require the action of growth factors such as FGF2. One related study demonstrated no quantitative change in FGF2 mRNA expression in sensory epithelia after ototoxic damage when compared to untreated chicks (Pickles and van Heumen, 1997). It is important to note that this study did not specifically check localized expression in the supporting cells.

The current understanding of FGF2’s role in auditory trauma is still limited. Further investigation is required to determine the degree of FGF2’s protective or restorative effects in hearing as well as the mechanisms that may drive this function. In the meantime, FGF2 has been studied extensively in other auditory contexts, particularly in TM repair.

## FGF2 for Tympanic Membrane Regeneration

The TM is a thin (~0.1 mm) layer of tissue which separates the external and middle ear. In the normal hearing process, the TM receives sound vibrations and transmits them to the auditory ossicles. The TM also serves as a barrier protecting the middle ear space from water, bacteria, or other foreign substances. Rupture of the TM can occur due to middle ear infections, barotrauma, loud sound exposure, or severe head trauma. While most TM perforations heal spontaneously within a few weeks, failure of healing can result in chronic perforation which can lead to hearing loss, infections, middle ear cholesteatoma, and other complications. Delayed healing may also necessitate surgical intervention for closure of the rupture. In recent years, alternative treatments for the repair of TM perforations have been explored including topically applied growth factors such as FGF2.

### Proposed Role of FGF2 in Tympanic Membrane Healing

The precise mechanisms by which FGF2 functions in human TM repair remains unclear, however, studies performed in animal models have provided insight into its potential roles. TM healing proceeds through three stages: inflammatory, proliferative, and remodeling (Somers et al., 1997). The inflammatory stage, which occurs 48–72 h post-injury, is characterized by swelling and a local exudative reaction composed of interstitial fluid, lymph, and blood (Wang et al., 2004; Santa Maria et al., 2010). The proliferative stage occurs 3–4 days post-injury during which FGF2 facilitates migration and proliferation of keratinocytes at the perforation border (Ishibashi et al., 1998). Additionally, FGF2 has been found to intensify epithelial mitotic activity and mediate the connective tissue reaction in the middle epithelial layer (Ishibashi et al., 1998). Through this process, an epithelial bridge is formed over the area of injury (de Araújo et al., 2014). In the final stage of healing, the outer epithelial and inner mucosal layer thins, and fibroblasts in the middle layer become small and flattened (de Araújo et al., 2014). Levels of FGF2 are increased at the site of epithelial proliferation on day 3 post-injury with peak levels seen on day 5 post-injury (Werner et al., 1992; Ishibashi et al., 1998). When used therapeutically in rats (Vrabec et al., 1994), chinchillas (Kato and Jackler, 1996), and guinea pigs (Fina et al., 1991, 1993; Ozkaptan et al., 1997), animals treated with FGF2 had a shortened TM healing time and improved closure rate when compared with controls. The acceleration of closure time may be due in part to FGF2’s vasodilatory effects which stimulate increased local blood flow (Mondain and Ryan, 1994).

### Clinical Studies of FGF2 for Tympanic Membrane Regeneration

Building upon promising pre-clinical findings, several studies have been performed exploring the therapeutic potential of FGF2 for TM regeneration in patients (Table 4). Twelve of the identified studies did not have a control group for comparison (Klejbor et al., 2006; Hakuba et al., 2010, 2013, 2015a,b; Lou et al., 2014, 2015a,b; Lou X. et al., 2015; Acharya et al., 2015; Omae et al., 2017; Kanemaru et al., 2018; Kanemaru et al., 2021). The closure rate of TM perforations in these studies was reported between 62 and 100%.

**Table 4:** Clinical studies of FGF2 in tympanic membrane repair.

Study	Country	N	Study design	Treatment groups	Frequency	Outcome	Effect
Hakuba et al. (2003)	Japan	14	Prospective clinical study	Observation and 0.2 ml of FGF2 (100 μg/ml)	Once a day for 3 days, follow up 1–2 weeks after treatment. Repeated treatment rounds until complete closure of TM.	Closure rates in FGF2 and observation groups were 100% and 40%, respectively. PTA improved by 13 dB following FGF2 treatment.	Improved TM closure rate
Hakuba et al. (2010)	Japan	87	Prospective clinical study	0.1 mL of FGF2 (100 μg/ml) FGF2 *via* atelocollagen	Applied once with a follow up at 3 weeks after treatment. Repeated treatment rounds until complete closure of TM.	Complete closure rate was 92.0%. PTA improved by 14 dB following FGF 2 treatment.	N/A
Kanemaru et al. (2011)	Japan	56	Randomized control trial	Observation and 5–30 μg of FGF2 from 100 μg/ml solution *via* gelatin sponge	Applied once with a follow up at 3 weeks after treatment. Repeated treatment rounds (up to 4 rounds) until complete closure of TM.	Closure rates in FGF2 and observation groups were 98.1% and 10%, respectively. Improvement of 22 dB in PTA at low frequencies, and 32 dB at high frequencies following FGF2 treatment.	Improved TM closure rate
Lou et al. (2012)	China	147 (ears)	Prospective clinical study	FGF2 *via* Gelfoam	Twice daily until complete closure (up to 1 month)	Closure rates were 98.6%, 97.6%, 96.3%, and 100%, respectively, at following treatment initiation times:<3 days, 4–7 days, 8–14 days, and 2–4 weeks after injury. No significant difference in closure rates or healing time between the three groups. Significant improvement in air-bone gap following perforation closure.	N/A
Lou (2012)	China	94	Prospective, randomized, controlled trial	Observation; FGF2, and FGF2 *via* Gelfoam (4–5 drops of 21,000 IU/5 ml)	Daily until complete closure (up to 2 weeks)	Closure rates in direct FGF2 application, FGF2 *via* Gelfoam, and observation groups were 100%, 97%, and 55%, respectively. Significant increase in closure rates and decrease in closure time in FGF2 treated groups when compared to the observation group. No significant difference in closure rate or healing time between the two FGF2 treated groups.	Improved TM closure rate and time
Zhang and Lou (2012)	China	104	Prospective, non-blinded, controlled study	Observation and drops of FGF2 (21,000 IU/5 ml)	Daily until complete closure (up to 3 months)	Significant increase in closure rates at 3 months in FGF2 group (100%) when compared to observation group (77%). Significantly shorter closure time in the FGF2 group (12.6 ± 1.2 days) when compared to the observation group (43.1 ± 2.5 days). Mean PTA improvement after 3 months was 12 dB for the FGF2 group, and 12 dB for the control group.	Improved TM closure rate and time
Lou and Wang (2013)	China	58	Prospective, sequential allocation, three armed, controlled clinical study	Observation, ~0.25 ml of FGF2 solution (21,000 IU/5 ml), and edge approximation	Daily until complete closure (up to 6 months)	Significantly higher closure rate in FGF2 group (100%) when compared to edge-approximation group (60%) or observation group (56%). Significantly shorter closure time in the FGF2 group (12.4 ± 3.6 days) when compared to edge-approximation group (46.3 ± 8.7 days) or observation group (48.2 ± 5.3 days).	Improved TM closure rate and time
Hakuba et al. (2013)	Japan	116	Retrospective cohort study	0.1 ml of FGF2 solution	Once	TM closure was achieved in 62% patients after 1-year. Epithelial pearl formation was observed in 5% of patients with an average onset time of 7.3 months.	N/A
Lou et al. (2014)	China	126	Prospective clinical study	0.1–0.15 ml (lower dose) or 0.25–0.3 mL (higher dose) of FGF2 (21,000 IU/5 ml)	Daily until complete closure (up to 3 months)	Closure rate was 92% in low dosage and 100% in high dosage for large perforations. The lower dosage group had a significantly shorter closure time compared with the higher-dose group (7.9 ± 2.5 vs. 12.5 ± 6.5, respectively) for medium sized perforations. The dose of FGF2 did not significantly affect the closure rate of large-sized perforations (92% vs. 100%) or mean closure time (11.8 ± 4.7 vs. 15.1 ± 6.1).	N/A
Lou Z. et al. (2016)	China	29	Prospective clinical study	Observation; 0.1–0.15 ml of FGF2 (21,000 IU/5 ml)	Once daily until complete closure (up to 6 months)	Closure rates at 6 months in FGF2 and observation groups were 91.7% and 52.9%, respectively.	Improved TM closure rate
Acharya et al. (2015)	Australia	13	Prospective cohort study	FGF2 *via* Gelfoam	Once	The overall closure rate was 83% and hearing improvement was observed in 80% of successfully treated cases. Mean four-frequency average air conduction threshold improvement in patients with TM closure was 9 dB, which was a significant improvement.	N/A
Zhengcai-Lou et al. (2016)	China	86	Prospective clinical study.	Observation; 0.1–0.15 ml of FGF2 (21,000 IU/5 ml), and EGF groups	Daily until complete closure (up to 3 months)	No significant difference in closure rates between EGF (86.2%), FGF2 (89.3), and observation (72.4%) groups. EGF and FGF2 groups had significantly shorter closure time when compared to the observation group. Mean PTA improvement after 3 months was 13 dB for the EGF group, 13 dB for the FGF2 group, and 13 dB for the observation group. Differences in hearing improvement among the groups were not statistically significant.	Improved TM closure rate
Lou et al. (2015b)	China	99	Retrospective cohort study	0.1–0.15 ml of FGF2 (21,000 IU/5 ml)	Once daily until complete closure (up to 6 months)	The closure rate was 92.9% at 6 months and the mean closure time was 10.59 ± 6.81 days.	N/A
Lou and Wang (2015)	China	93	Prospective and randomized clinical study	Observation and 0.2–0.25 mL of FGF2 (21 000 IU/5 ml) groups	Daily until complete closure (up to 6 months)	Significant increase in closure rates in FGF2 treated groups (97.8%) when compared to the observation group (82.5%). Significant decrease in closure time in FGF2 groups (12.5 ± 3.4 days) when compared to the observation group (34.0 ± 5.9 days). No difference in closure rate, but significantly shorter closure time between treatment initiation at ≤3 days compared to >3 days.	Improved TM closure rate and time
Lou et al. (2015a)	China	18	Prospective clinical study	0.10–0.15 ml of FGF2 (21000 IU/5 ml)	Daily until complete closure (up to 6 months)	The closure rate was 94.1% and the average closure time was 28.4 ± 10.9 days. Statistically significant improvement in the air-bone gap in patients who achieved TM closure. Bone conduction thresholds improved in 5 cases with mixed hearing loss.	N/A
Hakuba et al. (2015b)	Japan	10	Single arm and exploratory clinical trial	0.1–0.2 ml of FGF2 *via* atelocollagen sponge/silicon membrane	Once	The closure rate was 81.8% at 1-year postoperative follow-up. Mean improvement in PTA was 9 ± 6 dB in six cases that achieved complete TM closure.	N/A
Hakuba et al. (2015a)	Japan	153	Retrospective cohort study	0.1–0.2 ml of FGF2 (100 μg/ml) *via* atelocollagen/silicon membrane	Treatment round with follow up after 2–3 weeks. Repeated rounds of treatment until complete closure (up to one-year)	66.0% of patients achieved complete closure, 19.6% of patients had residual pinhole perforations (<1 mm diameter), and 14.4% of patients had larger residual perforations.	N/A
Lou Z.-C. et al. (2016)	China	185	A prospective, quasi-randomized, controlled clinical study	Observation; Gelfoam; 0.15–0.2 ml of FGF2 solution (21,000 IU/5 ml); and ofloxacin eardrops	Once daily until complete closure (up until 6 months)	No significant difference in closure rates between observation (82.2%), FGF2 (93.2%), gelfoam (85.7%), and ofloxacin (92.3%) groups. Significantly decreased mean closure time for all treatment groups when compared to observation. The mean closure times were 25.6 ± 13.32, 12.3 ± 8.15, 14.3 ± 5.44, and 13.97 ± 8.82 days for the observation, FGF2, Gelfoam, and ofloxacin groups, respectively.	Improved TM closure time
Omae et al. (2017)	Japan	11	Prospective, multicenter, open-label, single-arm and exploratory clinical trial	5–30 mg of FGF2 (100,000 μg/ml) *via* Gelfoam	Once	TM closure and hearing improvement was achieved in 88.9% of patients at the 12-week time point. Improvement in the air-bone gap and mean air conduction threshold was observed with FGF2 treatment. Mean bone conduction thresholds at 0.5, 1, and 2 kHz were significantly lower than those at baseline in FGF2 treated patients. Speech recognition threshold and maximum speech discrimination score both significantly improved following FGF2 treatment.	N/A
Jin et al. (2017)	China	138	Prospective, randomized	Observation; Gelfoam; and 2–3 drops of FGF2 (21,000 IU/5 ml) *via* Gelfoam	Every other day until complete closure (up to 6 months)	Significant increase in closure rates in the FGF2 *via* Gelfoam (97.9%) and Gelfoam (89.8%) groups when compared to the observation group (70.7%). Significant decrease in closure time in the FGF2 *via* Gelfoam group (15.7 ± 5.1) when compared to Gelfoam (24.8 ± 4.9) and observation (35.7 ± 9.2 days) groups. PTA improvement after 6 months was 13 dB in the FGF-2 *via* Gelfoam group, 14 dB in the Gelfoam alone group, and 14 dB in the observation group; these differences were not significant.	Improved TM closure rate and time
Lou and Lou (2017)	China	180	Prospective, randomized, clinical trial	Observation, EGF, 0.1–0.15 ml of FGF2 (21,000 IU/5 ml), and 0.3% ofloxacin eardrops	Once daily until complete closure (up to 6 months)	No significant difference in closure rates between EGF (91.11%), FGF2 (93.18%), ofloxacin (95.65%), and observation (82.22%) groups. Significantly increased closure time in observation group when compared to treatment groups. The mean PTA improvement after 6 months was 11 dB for the EGF group, 11 dB for the FGF-2 group, 11 dB for the ofloxacin group, and 9 dB for the observation group. The improvement rates between the groups were not statistically significant.	Improved TM closure rate
Zheng-Cai and Zi-Han (2018)	China	134	Prospective, randomized, controlled trial	Observation and ~0.15 mL of FGF2 (21,000 IU/5 ml)	Once daily until complete closure (up to 6 months)	Significant increase in closure rates in the FGF2 group (95.5%) when compared to observation group (73.4%). Additionally, the FGF2 group (11.9 ± 3.1 days) had a significantly shorter closure time when compared to the observation group (52.6 ± 18.1). No significant difference in PTA improvement between the two groups.	Improved TM closure rate
Kanemaru et al. (2018)	Japan	45	Controlled, pilot study	FGF2 *via* gelatin sponge	Applied once with a follow up at 3 weeks after treatment. Repeated treatment rounds (up to 4 rounds) until complete closure of TM.	Complete closure of the TM was achieved in 91% of patients. Improvement in average hearing levels and air-bone gap when compared to the historical control group.	N/A
Santos et al. (2020)	USA	54	Randomized, double-blind, placebo-controlled phase 2 clinical trial	Placebo (sterile water) or 20 μg/0.2 ml FGF2 *via* gelatin sponge	Applied once with follow up at 3 weeks after treatment. Repeated treatment rounds (up to 3 rounds) until complete closure of TM.	No significant difference in closure rate between placebo (71.4%) and FGF2 (57.5%) treated groups. No significant difference in pure tone averages or word recognition scores between study groups.	No effect
Lou et al. (2021)	China	29	Prospective cohort control study	Myringoplasty; 0.1–0.15 ml of FGF2 (21,000 IU/5 ml)	Applied twice daily for 3 months to the TM	In patients with perforation secondary to COM, FGF2 treatment alone achieved an overall closure rate of 36% compared to 100% in all patients who underwent myringoplasty. The closure rate with FGF2 treatment was 66.7% in smaller sized perforations. No significant difference in PTA improvement between groups.	No effect
Kanemaru et al. (2021)	Japan	20	Multicenter, non-randomized, single-arm study	10–100 μg FGF2 *via* gelatin sponge	Applied once with follow up at 4 weeks after treatment. Repeated treatment rounds (up to 4 rounds) until complete closure of TM.	Total closure of TMP at 16 weeks was achieved in 75% of patients with the mean decrease in perforation size of 92.2%. There was a significant improvement in the air-bone gap with FGF2 treatment when compared to baseline. Air conduction threshold and air-bone gap significantly improved following FGF2 treatment. The speech recognition threshold significantly improved following FGF2 treatment.	N/A

*TM, tympanic membrane; PTA, pure tone average; IU, international unit; EGF, epidermal growth factor; COM, chronic otitis media*.

The remaining 14 studies compared patients treated with FGF2 to either those who had no intervention, placebo, or an alternative treatment (e.g., Gelfoam, ofloxacin eardrops, and myringoplasty). Many of these studies reported improved closure rates (Hakuba et al., 2003; Kanemaru et al., 2011; Lou Z. et al., 2016), shortened closure times (Lou Z.-C. et al., 2016; Zhengcai-Lou et al., 2016; Lou and Lou, 2017; Zheng-Cai and Zi-Han, 2018), or both (Lou, 2012; Zhang and Lou, 2012; Lou and Wang, 2013, 2015; Jin et al., 2017) with FGF2 treatment when compared to observation. However, in a Phase II trial of 54 patients, no significant difference in TM closure rates or hearing improvement were observed when comparing FGF2 treatment with placebo (Santos et al., 2020). Treatment with FGF2 compared with Gelfoam alone significantly shortened time to closure but had no significant difference in closure rate (Lou Z.-C. et al., 2016). In a different study, a comparison between Gelfoam alone and Gelfoam with FGF2 showed that co-application with FGF2 significantly improved closure rates and decreased closure time (Jin et al., 2017). No significant differences in closure rates or time to closure were observed between patients treated with FGF2 and ofloxacin drops (Lou Z.-C. et al., 2016; Zheng-Cai and Zi-Han, 2018). When compared with traditional myringoplasty in patients with TM perforation secondary to chronic otitis media (COM), FGF2 treatment had a much lower overall closure rate (36 and 100%, respectively; Lou et al., 2021). However, when grouped by size, FGF2 treatment had a higher closure rate in smaller perforations when compared to medium-sized perforations in patients with COM (66.7% vs. 0%, respectively; Lou et al., 2021).

## Consideration of FGF2 Activity in Tumors Related to Hearing Loss

FGF2 has been found to have tumor-promoting effects which is not unexpected given its classification as a growth factor. Conversely, studies have also shown that FGF2 may suppress tumor growth. In the auditory system, sporadic vestibular schwannomas (VS) are the most common tumors of the cerebellopontine angle, and often present with sensorineural hearing loss (SNHL; Mahaley et al., 1990). It was previously assumed that SNHL due to VS was mediated solely by tumor compression of the cochlear nerve, however, it is now recognized that VS-secreted factors potentiate damage to the auditory system (Dilwali et al., 2013, 2015; Wu et al., 2021). FGF2 has been identified as a VS-secreted factor that may serve a protective role against SNHL (Dilwali et al., 2013). Specifically, VS-secreted FGF2 levels had a negative correlation with the degree of SNHL (Dilwali et al., 2013, 2015) because they were positively correlated with word recognition scores, and negatively correlated with pure tone averages (Dilwali et al., 2015). These studies suggest that FGF2 may have protective effects on hearing. However, the dichotomous role of FGF2 in tumors requires further investigation.

## Discussion

Knowledge of the role of FGF2 in auditory development and interest in its potential as a therapeutic agent has advanced considerably in the past decades. In this review, we comprehensively summarized: (1) the expression pattern and activity of FGF2 during inner ear development and maintenance in vertebrate models; (2) the effect of FGF2 on proliferation and differentiation of inner ear cells *in vitro*; (3) the role of FGF2 as a preventive and curative agent in auditory damage; (4) regenerative function of FGF2 in TM; and (5) FGF2 activity in tumors related to hearing loss. These studies highlight the potential of FGF2 based therapies for hearing disorders. However, there are many unanswered questions prior to verifying FGF2 as a useful therapeutic for hearing loss. Therefore, future studies need to be conducted to fill these gaps in knowledge which are outlined below.

### Limitations of Preclinical Studies

#### Mammalian Studies

Both *in vitro* and *in vivo* experiments of induced auditory trauma with mammalian animal models (Table 3) have yielded variable results, calling for additional studies to define optimal treatment parameters prior to transitioning to clinical studies. Current *in vitro* evidence suggests that the stimulatory effect of FGF2 is concentration-dependent. The survival rate and length of mouse neurites were found to be directly correlated with the added concentration of FGF2 (Zhai et al., 2004). Treatment with a high concentration of FGF2 (500 ng/ml) had a stimulatory effect (Low et al., 1996) that was not seen with low concentration (10 ng/ml; Lou et al., 2014).

In existing *in vivo* studies, the degree of improvement with FGF2 varied between studies, partially owing to differences in the type of auditory insult (e.g., blast, noise, ototoxin, and compression). For studies that used noise-induced trauma, parameters such as the intensity (115–172 dB) and duration of exposure (4–5 h) varied significantly. Therefore, direct comparison even in these studies with the same mechanism of auditory trauma was challenging as more than one parameter was different. Two studies used the same noise parameters and differed only in route of FGF2 delivery (Zhai et al., 2002, 2004). In Zhai et al. (2002), FGF2 was delivered directly to the cochlea. In contrast, Zhai et al. (2004) delivered FGF2 at the same concentration (volume not specified) *via* intramuscular injection in the buttocks. The mean improvement in the ABR threshold was 27 dB for intramuscular delivery compared to 19 dB for intracochlear delivery (Zhai et al., 2002, 2004). Since the volume of drug was not specified in Zhai et al. (2004), it is difficult to conclude whether route or dosing led to the different outcomes. However, there was a uniform improvement of hearing threshold in all studies when FGF2 was given after an auditory insult (Zhai et al., 1997, 2002, 2004; Yin et al., 2002; Shi et al., 2003). This supports a rescue role for FGF2.

In contrast, in two studies in which FGF2 was given prior to noise-induced or ototoxic damage, there was no effect (Yamasoba et al., 2001; Wimmer et al., 2004). Considering the short biological half-life of free form FGF2 (less than 1 h; Edelman et al., 1993), its utility as a protective agent may be limited. In Wimmer et al. (2004), ototoxic damage was induced over a period of 5 days, and in Yamasoba et al. (2001), noise-induced trauma was started 4 days after FGF2 delivery. It may be that these studies missed the treatment window for FGF2. The lack of consistency in auditory trauma parameters and FGF2 treatment between current pre-clinical studies remains a limitation. Nonetheless, the consistent findings when given after auditory damage support further development as a rescue agent.

Interestingly, when provided as a genetic construct, FGF2 treatment was found to have both protective and rescue effects (Yin et al., 2002; Shi et al., 2003). Gene therapy using an FGF2 genetic construct can be tailored for precise FGF2 dosing and targeting. In the case of auditory trauma, such a genetic approach is expected to provide long-term and stable expression of FGF2 in specific target cells such as hair cells or SGNs. This long-lasting expression may underly the added protective effect seen with this approach.

#### Non-mammalian Studies

Studies performed in non-mammalian models have provided insight into FGF2 expression levels and distribution following induced auditory trauma. However, studies have not been consistent in their findings. It has been suggested that these inconsistencies may be a result of variable durations following induced auditory or ototoxic damage at which FGF2 levels were analyzed. For example, Umemoto et al. (1995) examined protein expression 1 day after noise exposure. Lee and Cotanche (1996) and Pickles and van Heumen (1997) measured mRNA and protein levels 2 days following noise or ototoxic damage. Additionally, the method by which auditory damage was induced varied between studies. Given that ototoxic damage works over a longer time period than acoustic damage, it may be that even if there were changes in FGF2, this would not be reflected in mRNA levels. Additionally, studies demonstrating no quantitative changes of FGF2 did note a redistribution of FGFR1, a high-affinity receptor for FGF2, from hair cells to supporting cells following the damage. Therefore, it may be that FGF2 protein was locally increased to promote regeneration of avian hair cells *via* stimulation of FGFR1. Another hypothesis is that FGF2 may be involved in stimulating precursor cell differentiation in inner ear epithelia since stimulation of postnatal avian inner ear epithelia by FGF2 did not lead to a direct increase in cell proliferation, but rather blocked mitogenesis (Oesterle et al., 2000).

Future studies can help clarify whether differences between the regenerative capacities of the avian and mammalian inner ear reflect, at least in part, variations in cellular patterns and timing of FGF2 expression. Similar experiments are needed in mammalian models to define FGF2 expression levels at different time points following auditory trauma, as these experiments may provide insights into a possible therapeutic window to repair damaged inner ear cells.

### Optimization of FGF2 Treatment for Clinical Studies

While clinical studies have revealed the therapeutic potential of FGF2 in TM repair, there remains uncertainty around the ideal dose, timing, duration, method of delivery, and patient selection for enhanced therapeutic effect.

One study examined two different doses of FGF2 for healing of medium (1/8 to 1/4 of TM) and large (greater than 1/4 of TM) perforations (Lou et al., 2014). They found that the lower dose (0.1–0.15 ml of 21,000 IU/5 ml recombinant bovine FGF2 solution) had a significantly shorter closure time for medium-sized perforations when compared to the higher dose (0.25–0.3 ml FGF2 solution). The FGF2 dose had no significant impact on medium-sized perforation closure rate, or large-sized closure rate and repair time. It may be that continuous application of a higher dose of FGF2 inhibits collagen synthesis in the fibrous layer, thereby prolonging time to closure (Ryan and Baird, 1993; Lou, 2012; Lou et al., 2014). However, several factors such as age, gender, duration of injury, and cause of injury may also impact healing time and rates.

With regards to the timing of FGF2 administration, Lou and Wang (2015) have shown that mean closure time was significantly shortened when FGF2 was applied 3 days after injury, which corresponds to the proliferative stage of healing. Two additional studies have compared initiation of FGF2 treatment at specified times of 3, 4–7, 8–14, and >15 days following injury (Lou et al., 2012, 2015b). There was no statistically significant difference in closure rate or healing time between these groups, although those treated in the 8–14-day window after injury had the shortest time to closure (Lou et al., 2012, 2015b). Even though these studies indicate that the best commencement time of application may be after the inflammatory stage of wound healing, further research is needed to clarify the best time to apply FGF2 for TM perforation.

No consensus has been reached on the preferred duration of FGF2 treatment. Many studies have administered FGF2 daily until the TM perforation is completely healed (Lou et al., 2012, 2014, 2015a,b, 2021; Lou, 2012; Zhang and Lou, 2012; Lou and Wang, 2013, 2015; Lou Z. et al., 2016; Lou Z.-C. et al., 2016; Zhengcai-Lou et al., 2016; Lou and Lou, 2017; Zheng-Cai and Zi-Han, 2018). However, this approach can be inconvenient for patients with prolonged healing times. Furthermore, prolonged treatment may also trigger otorrhea or cause excess moisture buildup which can impair healing and damage surrounding tissue (Okan et al., 2007). Previous studies with other growth factors have also found that large doses or long-term application can result in reperforation of the eardrum or formation of middle ear cholesteatoma (Hennessey et al., 1991; Dvorak et al., 1995). An alternative approach has been to administer FGF2 every other day and this has been found to reduce otorrhea while allowing FGF2 to exert a continual effect (Jin et al., 2017).

Various routes of FGF2 delivery have been studied including direct application or administration *via* biomaterials. It has been hypothesized that biomaterials could serve as scaffolds for epithelial migration and allow for sustained release of FGF2. Furthermore, biomaterial patches may even serve as protection from infection during the TM healing process (Kanemaru et al., 2011). Several studies administered FGF2 with either atelocollagen or Gelfoam with beneficial effects on TM healing (Hakuba et al., 2010, 2013, 2015a,b; Kanemaru et al., 2011, 2018, 2021; Lou et al., 2012; Lou, 2012; Acharya et al., 2015; Jin et al., 2017; Omae et al., 2017). However, when FGF2 administered directly was compared to FGF2 provided *via* Gelfoam, no significant difference was observed in closure rate or healing time (Lou, 2012). The benefits of FGF2 *via* biological material patching need to be further validated in studies involving proper control groups.

The conflicting results of FGF2 therapeutic efficacy from recent studies (Santos et al., 2020; Lou et al., 2021) may be related to differences in patient selection criteria. For example, Santos et al. (2020) included patients with prior surgically repaired ears. Previous studies have suggested that surgical tympanoplasty can damage progenitor cells at the umbo or annulus of the TM which can inhibit healing (Kanemaru et al., 2011; Hakuba et al., 2013). Even so, Santos et al. (2020) did not see a difference in response to FGF2 when comparing surgical and non-surgical ears in their study group. Furthermore, Lou et al. (2021) found that the FGF2 application for TM perforation due to COM was not effective. This suggests that FGF2 may have an improved effect in TM perforation secondary to traumatic perforation as opposed to other causes. In contrast, Kanemaru et al. (2018) found FGF2 treatment to be beneficial even in patients with TM perforation who had cholesteatomas, tumors, and severe calcification. While other treatment variables were not constant between these studies, they do highlight that FGF2 treatment may have improved efficacy in a more restrictive patient subgroup.

There remain large gaps in understanding of the optimal treatment regimen of FGF2 for TM repair. However, current evidence suggests that treatment initiation 3 days following TM injury, and an every other day dosing strategy might be a promising starting point. Furthermore, restriction of patient populations to those with traumatic perforations in non-surgically repaired ears may show the most benefit. Early evidence does not support a role for biomaterials. Future studies using well-defined patient populations with a longer period of follow-up will be useful in determining these treatment parameters.

### Implications in Hearing Loss

The link between middle- and inner-ear studies of FGF2 has not been explored in depth. Early evidence suggests potential FGF2 penetration into and activity within the inner ear even when applied to the middle ear. Damage to the TM is often reflected on an audiogram by an increase in the air-conduction threshold with an associated gap between air and bone conduction (air-bone gap). Repair of the TM is expected to improve the air conduction threshold, and thereby close the air-bone gap. Nearly all the clinical studies of FGF2 for TM repair reported improvements in PTA air conduction and air-bone gap in patients who achieved TM closure. Two studies, however, also reported improvements in bone conduction thresholds, which they attributed to improved sound transmission due to TM repair (Lou et al., 2015a; Omae et al., 2017). While improvements in bone conduction thresholds have been reported after addressing the cause of conductive hearing loss (Vijayendra and Parikh, 2011), bone conduction thresholds are typically thought to reflect inner ear processes. Therefore, the clinically improved bone conduction thresholds may, at least partly, be a result of FGF2 activity in the inner ear. This is not implausible given the pre-clinical studies outlined previously which support a rescue and proliferative effect of FGF2. Although FGF2 was applied to the middle ear in clinical studies of TM repair, some amount may have penetrated the inner ear, e.g., *via* the round window membrane, allowing for localized activity. This potential therapeutic effect in hearing is in line with previous findings of elevated levels of vestibular schwannoma-secreted FGF2 being associated with better hearing (Dilwali et al., 2013, 2015).

Hearing loss is the most common sensory deficit worldwide, and the burden of disability is projected to increase in the coming years (Sheffield and Smith, 2019). There are no approved pharmacological therapies for hearing loss. Therefore, the search for novel treatments for hearing loss is an important endeavor. Overall, the existing evidence reveals a therapeutic role of FGF2 in auditory disorders, especially in TM regeneration. Early studies suggest the benefit of FGF2 for TM repair in specific patient populations. However, additional focus must be placed on optimizing treatment strategy and defining therapeutic window. The relationship between FGF2 and sensorineural hearing is still unclear. Existing pre-clinical evidence suggests a therapeutic effect conferred by FGF2 treatment, but the mechanism of this effect is largely unknown. Future studies investigating the link between FGF2 and sensorineural hearing are needed to fill current gaps in knowledge and translate them to clinical studies. These studies may help accelerate the discovery of novel treatment approaches for a patient population that has few non-surgical options.

## Author Contributions

MJ and VS wrote the manuscript. KB prepared all the tables. VS prepared the figure. KS conceived, designed, and supervised the manuscript writing and editing. All authors contributed to the article and approved the submitted version.

## Conflict of Interest

The authors declare that the research was conducted in the absence of any commercial or financial relationships that could be construed as a potential conflict of interest.

## Publisher’s Note

All claims expressed in this article are solely those of the authors and do not necessarily represent those of their affiliated organizations, or those of the publisher, the editors and the reviewers. Any product that may be evaluated in this article, or claim that may be made by its manufacturer, is not guaranteed or endorsed by the publisher.
